# Supplementary material for: Polyherbal Combinations Used by Traditional Health Practitioners against Mental Illnesses in Bamako, Mali, West Africa
Source: Plants (Basel). 2024 Feb 4;13(3):454. doi: 10.3390/plants13030454 (PMC10857219; doi:10.3390/plants13030454)
Supplement: Supplementary file 1 [file plants-13-00454-s001.zip › plants-2847409-supplementary.pdf]

Appendix A

Questionnaire for interviewing healers

Identity of the healer

Name: Gender: Age: Ethnic group:

Village of current residence: Village of Origin: Town:

Marital status:

Religion:

Number of wives: Number of children: Number of boys: Age of 1st boy:

Assistants: □ Children □ Wife □ Parents □ Other (specify):

Occupation:

Primary source of income: □ Healing □ Farming □ Other (specify):

Other role in the village:

Diseases healing (five max):

How many mental patients do you receive per year?

Do you hospitalize?
□ Yes
□ No

Is there inpatient right now? □ Yes □ No

If yes, how many:

Origin of knowledge: □ Family □ Learning □ Revelation □ Other (specify):

Does your master live: □ Yes □ No

Are you trying to transfer your traditional herbal plant knowledge? □ Yes □ No

If yes, to whom?

□ Son □ Daughter □ Other relatives (specify) □ Others (specify)

If no, why?

Do you collaborate with the doctors of conventional medicine? □ Yes □ No

If yes, how:

□ Reference of patients □ Exchange of information's □ Others (specify)

If no, why?

Traditional interpretations of mental illnesses

- What are the different kinds of mental illnesses according to you?
- What are the causes of each kind of these illnesses?
- How do you make the diagnosis of these illnesses?

Methods and therapeutic tools

How you treat this illness?

□ Plants
□ Sacrifice
□ Incantations
□ Massage
□ Physical
□ Restraint
□ Flagellation
□ Quran
□ Conventional medicine

What are the plants that you use to treat these illnesses?

Plant

Local name: Banmanankan: Another dialect (specify):

Scientific name: Species: Family:

Parts used:
□ Leaves
□ Roots
□ Bark of roots
□ Bark of trunk
□ Flowers
□ Fruits

□ Whole plant
□ Other (specify):

Collection period:

□ Rainy season
□ Warm season
□ Cold season
□ Any season

Collection time:

□ Morning
□ Afternoon
□ Evening
□ Night

Appropriate equipment for collection:

Rituals associated with the collection of these parts:

Prohibitions related to the collection of these parts:

Methods of preparation

Technique of preparation used:

Quantity of product:

Water quantity:

Duration of preparation:

Another product associated with the plant:

Rituals related preparation:

Prohibitions related preparation:

Modes of administration

□ Drinking
□ Body bath
□ Steam bath
□ Inhalation
□ Fumigation
□ Others (specify):

Quantity:
□ Clueless
□ a pinch two fingers
□ a pinch three fingers
□ a pinch four fingers
□ a pinch five fingers
□ Others (specify):

Time of administration:

□ Morning
□ Midday
□ Evening
□ Night

Duration of treatment:

Precautions:

Side effects associated with the use of the plant:

If side effects, what do you do?:

Considered indications: □ Pregnancy □ Lactation □ Others (specify):

Rituals related administration:

Prohibitions related administration:

Place of harvest of the plant: where you find this plant?

□ In the district
□ In the region
□ In another region of Mali (specify):
□ In another country (specify):

□ Next to the river
□ Next in the hills
□ In shady area
□ Under trees
□ Everywhere

Safeguarding activities of this plant:

□ Culture training
□ Rationing its use
□ Collection
□ Storage
□ Packaging
□ Parts used

Prior Informed Consent

I, the undersigned………………………………………………… traditional healer have read and understood the objective of the study on the plants used in the treatment of mental diseases and the use of information that I give.

I agree to participate in this study.

(Signature)

The traditional healer Date
